# Electrical Conductance Device for Stent Sizing

**DOI:** 10.3389/fphys.2019.00120

**Published:** 2019-02-26

**Authors:** Ghassan S. Kassab

**Affiliations:** California Medical Innovations Institute, San Diego, CA, United States

**Keywords:** minimum stent area, lumen sizing, diameter, conductance catheter, drug eluting stents

## Abstract

The minimum stent area (MSA) has been clinically established as a significant predictor of restenosis, thrombosis, and ischemia using intra-vascular ultrasound (IVUS). Unfortunately, IVUS measurements are far from routine because of significant cost of IVUS, the training required, the subjectivity of image interpretation and the time added to the procedure. The objective of this study is to verify the accuracy of a conductance catheter for stent sizing. Here, we introduce an easy and entirely objective device and method for real time determination of MSA. A 10 kHz, 35 μA rms current is passed through the external electrodes of an intravascular catheter while the conductance is measured across a separate set of electrodes. Both phantom and *ex vivo* validations of metal stent sizing in five porcine carotid arteries were confirmed. The accuracy of the measurements were found to be excellent in phantoms (root mean square, rms, of 3.4% of actual value) and in *ex-vivo* vessels (rms = 3.2% of measured value). An offset of conductance occurs when a conductive metal stent (e.g., bare metal stent) is deployed in the vessel, while the slope remains the same. This offset is absent in the case of drug eluting stent where the metal is coated (i.e., insulated) or non-metal bioresorbable stent. The present device makes easy, accurate and reproducible measurements of the size of stented blood vessels within 3.2% rms error. This device provides an alternative method to sizing of stent (i.e., MSA) in real-time without subjective interpretation and with less cost than IVUS.

## Introduction

Many studies have shown that the minimum stent area (MSA) is an important predictor of prognosis and later events such as restenosis, thrombosis, myocardial ischemia, and so on (Kasaoka et al., 1998; Wu et al., 2003; Fujii et al., 2004, 2005). This observation has led to the notion of “bigger is better” (Di Mario and Karvouni, 2000). The limit to such larger size is, of course, vessel injury, dissection and edge stenosis when the vessel is overly distended. Hence, it is clinically important to determine the MSA accurately.

Angiography, intra-vascular ultrasound (IVUS) and optical coherence tomography (OCT) are techniques than can be currently used to determine the size of a vessel after stenting. A difficulty with angiography is the poor resolution with the two-dimensional view, typically obtained from a single x-ray projection. Furthermore, trapping of contrast agent near the stent lattice often creates hazing or fuzziness in the angiogram, which further reduces the accuracy of measurement (Ziada et al., 1997; Grewal et al., 2001). IVUS, on the other hand, is more accurate and reliable. Other factors, however, limit its routine clinical use. The cost of IVUS (device and console), the significant training required, and the subjectivity of image interpretation have significantly limited its usage to less than 20% of routine procedures despite being on the market for over 20 years. Finally, OCT is primarily used as a research tool for high spatial resolution images to assess the interaction of struts with vessel wall but has limited penetration and is even more expensive than IVUS. Hence, it is desirable to introduce easier, more cost effective and entirely objective tools for MSA measurements to improve clinical outcome.

Kassab (Kassab et al., 2005, 2009; Hermiller et al., 2011; Nair et al., 2018) introduced an impedance catheter and guidewires that allows real time vessel lumen sizing based on an electric impedance principle. These devices were validated in silico, *in vitro* and *in vivo* in swine (Kassab et al., 2005, 2009) and patients (Hermiller et al., 2011; Nair et al., 2018). As a proof of concept, we modify the catheter and technique of determining vessel size in the presence of a stent (typically a metal; either bare metal or drug coated). It is noted that contact of the impedance electrodes with bare metal stent (BMS) can cause electrical shorting of signal and significant resulting noise, which prohibits accurate measurements. Furthermore, the presence of a bare metal in the measurement field also affects the conductance and introduces an offset. The present study proposes solutions to overcome these issues. The major conclusion is that, with a small modification of the catheter, accurate measurements of MSA can be made with the current device. Since most stents used clinical are drug eluting (coated and hence electrically insulated), no modification of the device is needed as described below.

## Materials and Methods

### Design Modification of Impedance Catheter for Stent Sizing

**FIGURE 1:**
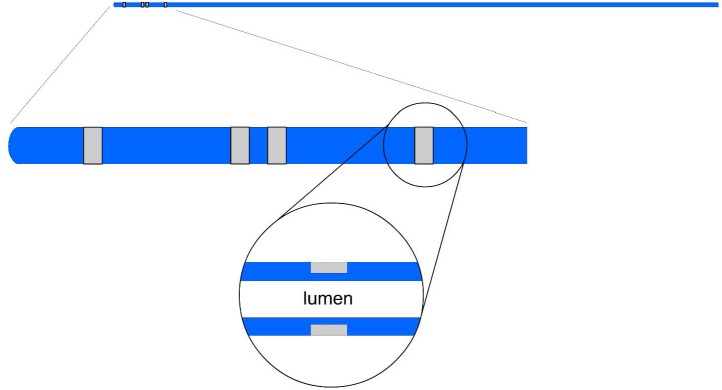
An illustration of an impedance catheter where the four electrodes are spaced at the tip (two inner and two outer electrodes) in the top panel; a zoom of the embedded portion of the electrode arrangement is shown in the middle panel; and a further zoom of the wire tunneling is shown in the lower panel.

The impedance catheters were similar to those used in previous studies (Kassab et al., 2005). Four holes were made in the catheter 5 mm, 9 mm, 10 mm, and 14 mm from the tip (i.e., 4-1-4 mm spacings). One insulated wire was threaded through each hole. The portion of the wire exposed through each hole was then stripped of its insulation and wrapped around the catheter. Previously, the four electrodes were exposed at the surface of the catheter where direct contact with stent was possible. In the present study, a design was proposed where grooves are made into the catheter such that the wires were made sub-surface as shown in Figure 1. This design decreases surface contact of wires or electrodes with the stent while allowing the necessary exposure for the conducting electrode in the measurement field.

The exteriorized portions of the four wires through the lumen were connected to an electronic conductance module constructed in our laboratory. This module drives a 10 kHz, 35 μA (root mean squared, rms) constant current between the two outermost electrodes and measures the resultant voltage between the two inner electrodes. The voltage detected, moderated in amplitude by the impedance change through the NaCl solution, has a frequency of approximately 10 kHz. The data acquisition rate has a frequency of 10 kHz, with current injected and detected voltage channels.

### Conductivity of Fluid and Stent

A calibration of known CSA was made in four acrylic tubes with sizes ranging from 2.5 mm to 5.0 mm in diameter. The catheter was placed in each of the four tubes. All four tubes were filled with either 0.45 or 0.9% NaCl solutions at room temperature. By using the voltage reading from the catheter, the conductivity, σ, of both NaCl solutions was determined by plotting the conductance, *G*, against *CSA/L* (*G = a.CSA/L* where L is the distance between the inner electrodes). The calibration was then repeated with a stainless steel Jostent (316L stent, Jomed) embedded within each tube. The conductance readings were plotted in order to determine the effect of stent.

### Stent CSA in Tygon Tubing

The CSA measurements were taken with the catheter placed in the lumen of each tube with known CSA containing a stent. The conductivity was determined for each solution as described above and the measured conductance was adjusted to accommodate for the offset caused by the stent. The CSA was then calculated using *CSA = G⋅L/s*.

### Stent CSA in *ex-vivo* Vessels

The lumped cylindrical model that relates the conductance, *G* (*G = I/V*, ratio of current to voltage), to the CSA as:

(1) G=σ ⋅ CSA/L

holds if the electrical current is insulated within the cylinder. It is known that the vessel wall and any surrounding tissue are conductive, however, and will cause an offset error from current leakage known as the effective parallel conductance, *G_p_*. Since *G_p_* is constant at any given position on a vessel, using two different concentrations of NaCl solutions will result in the desired relation as:

(2a) $$\mathrm{CSA(t)}=L[G_{2}(t)-G_{1}(t)]/[\sigma_{2}-\sigma_{1}]$$

where “1” and “2” refers to 0.45% and 0.9% NaCl solutions with specific conductivities σ_1_ and σ_2_. If we assume that the vessel has a circular cross-section, we can compute the diameter as D = (4CSA/π)^1/2^; namely,

(2b) $$D(t)={\left(\frac{4L}{\pi }\frac{\Delta G(t)}{\Delta \sigma }\right)}^{\frac{1}{2}}$$

where Δ represents the difference in a quantity for the two injections.

**FIGURE 2:**
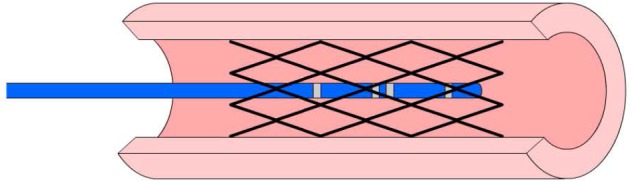
A schematic of an impedance catheter in the lumen of a stented vessel.

Five porcine carotid arteries were harvested from a local slaughter house to validate the stent sizing in *ex-vivo* vessels. The isolated carotid vessels were stored in 0.9% saline solution at 4°C. The carotid artery was cannulated with 6 Fr sheaths on both ends with one end connected to a pressure transducer. The stent was first deployed within the vessel and the catheter was then inserted through the sheath into the lumen of the artery as shown in Figure 2. The vessel was perfused with approximately 5 ml of 0.9% followed by 0.45% NaCl solutions. Each infusion was used to pressurize the vessel from 20 to 120 mmHg in increments of 20 mmHg for each solution. The pressurization to change the diameter of vessel was done by hand using a syringe connected to the second sheath. During pressurization, the vessel was placed under a camera to measure the outer diameter during inflation. By using the conductivities of different NaCl (0.45 and 0.9%) solutions with and without the stent and the conductance value for each solution, the CSA was determined by Eq. (2a) at each pressure. The diameter of the stented vessel was subsequently computed from Eq. (2b).

At the completion of experiment, a ring of the artery of 1 mm in thickness was obtained at the site of the detection leads of the catheter. A photo of the no-load (zero pressure) ring was taken under a dissection microscope and the no-load CSA of the vessel (CSA^nl^) was measured. Assuming incompressibility, the inner diameter of the vessel during pressurization was obtained from the outer diameter measurements described above. For a cylindrical vessel, the incompressibility assumption can be stated as:

(3) $$D_{i}=\sqrt{D_{o}^{2}-\frac{4CSA^{nl}}{\pi \;\lambda_{z}}}$$

where *D*_o_, *CSA^nl^* and *λ*_z_ are outer radii at the loaded state, wall area in the no-load state, and the axial stretch ratio, respectively. Hence, the lumen diameter or CSA (CSA = pD^2^/4. for a cylindrical vessel) determined from equation [3] was directly compared with the impedance measurements.

### CSA for Various Stent Metal Coils

In addition to actual stents, we used several wires to examine different stent metals with varying conductivities. Tungsten and stainless-steel type 316 wires of 0.25 mm diameter were shaped to a 2 cm long coil with approximately one full loop every 5 mm. The diameter of the coil was 3.5 mm for each type of metal and the experiments were repeated similar to those involving stents as described earlier.

### Drug Coated Stents *in vivo*

We also considered 3 drug eluting stents, DES (Xience, Abbott) deployed in swine (n = 2). The 3 mm stents were deployed at nominal pressure as suggested by manufacture’s chart as per our previous studies (Chen et al., 2011). These animals were used from other acute studies to maximize animal use. Briefly, normal male swine (60-65 Kg body weight) had expired DES deployed in each of three coronary arteries (RCA, LAD and LCx). The animal studies were approved by the Institutional Animal Care and Use Committee of Indiana University and complied fully with the Guide for the Care and Use of Laboratory Animals published by the National Research Council. After deployment of stent recommended diameter, IVUS was used to measure the diameter of the stent for comparison with the conductance measurements. The procedures and methods were the same as those reported in Ref. 9.

### Statistical Analysis

The relation between phantom (P) or optical (O) and impedance (I) diameter measurements were expressed by D_PorO_ = αD_I_ + β where α and β are empirical constants that were determined with linear least squares fit and a corresponding correlation coefficient R^2^. In a Bland-Altman scatter diagram, we plotted the percent differences between the two measurements of diameter $\left(\frac{D_{PorO}-D_{I}}{D_{PorO}}\times 100\right)$ against their means $\left(\frac{D_{PorO}+D_{I}}{2}\right)$. In the scatter diagram, the precison and bias of the method can be quantified. We also determined the root mean squares (rms) error to further assess the reliability of the technique.

## Results

### Conductivity of Saline and Stent

**FIGURE 3:**
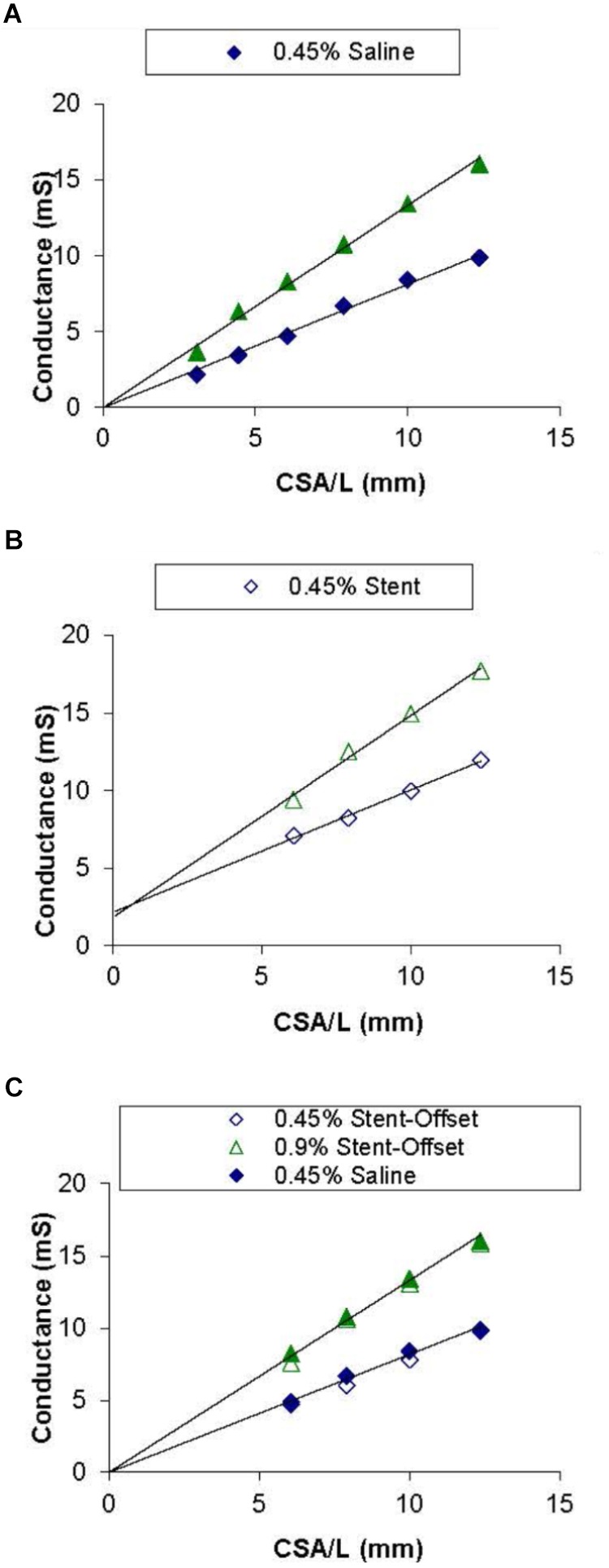
Saline and stent calibrations of 0.45 and 0.9% NaCl solutions. Graph **(A)** shows conductance values for each corresponding CSA in acrylic tubes filled with saline only. Graph **(B)** shows the values with the stent embedded in each of the tubes. Graph **(C)** shows both saline only and stent values with normalized offsets. Lines of best fit are shown in Graphs **(A)** and **(B)**.

Figure 3A shows the conductance measured by the catheter for each CSA in 0.45% and 0.9% NaCl solutions. A saline only calibration was used as a baseline run to show that the 0.9% saline has a higher (approximately 2 times) conductivity (slope) than the 0.45% saline solution. The NaCl measurements have a nearly zero offset (intercept) in the conductance readings. The calibration of NaCl in the presence of stent showed no change in conductivity (slope) of both solutions but resulted in an offset of about 3 mS for the stainless-steel stent as shown in Figure 3B. The two graphs were superimposed to demonstrate that when normalizing for the offset (i.e., subtract the intercepts from Figure 3A,B), the stent calibration is nearly identical to the saline calibration (Figure 3C).

The saline and stent calibrations were done with four catheters (3Fr and 4Fr) where the conductivity (slope) was unchanged with or without the stent. The conductance offsets of 3.2 ± 1.2 and 3.3 ± 0.72 for the 0.45% and 0.9% NaCl solutions, respectively, were significantly different from zero in the presence of stent (BMS).

### Stent CSA in Tygon Tubing

**FIGURE 4:**
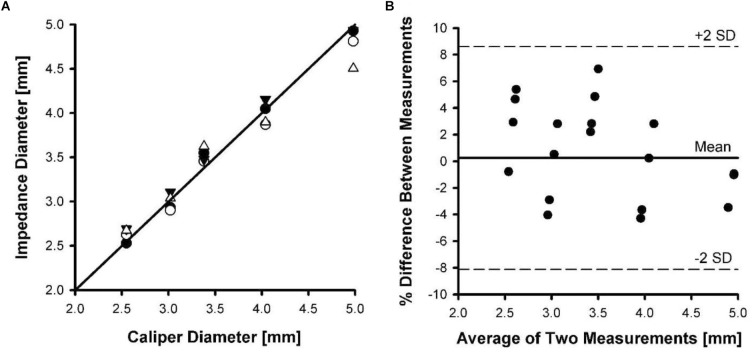
Phantom diameter measurements determined using impedance catheters versus a caliper. Four catheters were tested in five different tubing sizes. **(A)** A line of identity shown between caliper diameter and impedance diameter measurements. **(B)** A Bland-Altman plot of mean of diameter measurements versus percent difference in measurements.

Four catheters were placed in five different sized Tygon tubing with the deployed stent to determine the stented diameter. Although the diameter measurements were made with both NaCl solutions, the difference was not significant and the average from both solutions was used. Figure 4A shows the phantom diameter measured by the catheter as compared to the diameter measured with a caliper. To determine the agreement between the two methods, we made a Bland-Altman plot of the percent difference in diameters between the two methods against their mean values. Figure 4B shows the Bland-Altman plot where the mean and SD were found to be 0.25 and 4.2, respectively. The upper and lower dotted lines represent mean+2SD (8.6%) and mean-2SD (-8.1%), respectively. The rms error for the impedance measurements was 3.4% of the phantom diameter.

### Stent CSA in *ex vivo* Vessels

**FIGURE 5:**
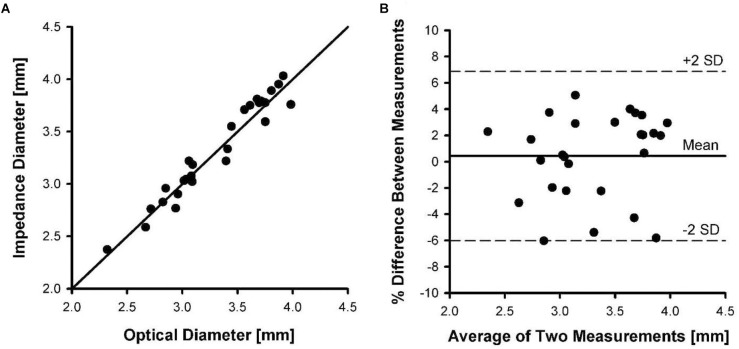
Inner diameters of five pig carotid arteries measured by impedance catheters versus diameters measured optically. Each artery was pressurized from 20 to 120 mmHg in increments of 20 mmHg during measurements. **(A)** The line of identity between optical diameter and impedance diameter measurements. **(B)** A Bland-Altman plot of mean of diameter measurements versus percent difference in measurements.

Figure 5A shows the relationship between carotid vessel diameters measured by impedance and optical methods. The correlation coefficient for the relationship between impedance and optical measurements was 0.946, with a slope and intercept of 1.01 and 0.034, respectively. Figure 5B shows the Bland-Altman plot where the mean and SD were found to be 0.37 and 3.4, respectively. The upper and lower dotted lines represent mean+2SD (6.8%) and mean–2SD (–6.0%), respectively. The rms error for the impedance measurements was 3.2% of the vessel diameter.

### CSA for Various Stent Metal Coils

The calibrations with metal (stainless steel and tungsten) coils show that the slopes remain the same (Table 1). There were also no differences in the offsets in relation to the NaCl (0.45 or 0.9%) solutions used as shown in Table 1. There were differences in the offsets, however, for the two metals. The stainless-steel coil offsets the conductance readings by about 4 mS in four catheters, while the tungsten coil records an offset of 45 mS. Hence, the more conductive tungsten material shows a significantly higher conductance offset (an order of magnitude) than stainless steel (p < 0.00001).

**Table 1:** The calibration slope and offset of the impedance catheter in NaCl and NaCl plus wire coil (stainless steel and tungsten).

Stainless Steel (SS)				
	0.45% NaCl	0.45%NaCl w/ SS	0.9% NaCl	0.9%NaCl w/ SS

Slope	1.1 ± 0.17	1.1 ± 0.11	1.9 ± 0.31	1.9 ± 0.25
Offset	-0.47 ± 0.18	3.8 ± 1.5	-1.1 ± 0.71	3.1 ± 1.8

**Tungsten (T)**				

	0.45% NaCl	0.45%NaCl w/T	0.9% NaCl	0.9%NaCl w/T

Slope	1.2 ± 0.28	1.1 ± 0.26	1.8 ± 0.41	1.9 ± 0.35
Offset	-0.78 ± 0.17	45 ± 5.6	-0.84 ± 1.6	46 ± 6.4


*The values are mean ± SD*.

### DES Stent CSA in *in-vivo* Vessels

In the stents used, the differences between the conductance catheter measurements and IVUS were 8.2%. We had also confirmed that the drug coated stents were not conductive and did not produce any offset as observed with BMS or stent metal coils. Hence, the two animal studies in 3 coronary arteries were purely confirmatory.

## Discussion

Although stenting reduces acute complications and restenosis as compared to balloon angioplasty, in stent restenosis (ISR) remains an important clinical problem (Chen et al., 2011). Studies suggest that a significant number of ISR lesions contain inadequately expanded stents (Schiele, 2005). Furthermore, stent underexpansion is a significant cause of failure after sirolimus-eluting stent (SES) treatment (Fujii et al., 2004). IVUS studies show that an MSA < 5 mm^2^ was the optimal threshold to predict target-lesion reascularization after treatment of de novo lesions with SES (Fitzgerald et al., 2000). MSA > 5 mm^2^ predicted long-term patency after treatment of de novo lesions with SES (Fujii et al., 2004, 2005).

Despite the utility of IVUS in the assessment of MSA, it has several shortcomings which limit its routine use in the catheterization laboratory. It requires advancement of a relatively expensive IVUS catheter connected to an expensive apparatus. It also adds longer procedure time and longer fluoroscopic time, increases the use of contrast material (Chen et al., 2011) and increases the risk of dissection, thrombosis, spasm and acute occlusion during catheter manipulation (Hausmann et al., 1995; Sonoda et al., 2004). Furthermore, it is not unusual that the relatively bulky IVUS catheter cannot be advanced across a lesion due to high grade stenosis, vessel tortuousity or calcification. Finally, since the impedance electrodes can be implanted within a workhorse guidewire (Nair et al., 2018), it is not necessary to change catheters such as with the use of IVUS.

The present device makes easy, accurate and reproducible measurements of the size of stented blood vessels within clinically acceptable error. This enables the determination of MSA with higher accuracy using previously published methods (Kassab et al., 2005, 2009; Hermiller et al., 2011; Nair et al., 2018). The present catheter addresses two key issues that are resolved by modification of the previous device design. First, the four electrodes were exposed at the surface of the catheter where direct contact with stent was possible (Kassab et al., 2005, 2009; Hermiller et al., 2011; Nair et al., 2018). In the present study, a design is implemented where grooves are made into the catheter such that the wires are made sub-surface as shown in Figure 1. This design decreases surface contact of wires or electrodes with the stent while allowing the necessary exposure for the conducting electrode in the measurement field.

The second issue addressed here relates to the offset creates by the presence of the stent in the vessel lumen. Previously, it was shown that sizing (cross-sectional area, CSA) is related to the ratio of change in conductance to change in conductivity (slope of the conductivity-conductance relation). Figure 3A shows the CSA/L-conductance relationship, which is expected to be linear with zero intercept (Eq. 1). The slope of Figure 3A corresponds to the conductivity s. Figure 3B shows the same relation in the presence of a stent. It is apparent that the slope of the curve remains unchanged but there is an offset that reflects the conductivity of the stent. Although the conductivity of the metal itself may vary, this will not affect the measurements as the slope which determines the CSA (Eq. 2) remains unchanged. In conclusion, the presence of a stent does not affect the sizing accuracy of the impedance catheter (errors < 5%) as shown in Figure 4, 5. The change of offset is inconsequential for the sizing utility of the conductance technology.

Although we used BMS and conductive stent metal to consider the worst-case scenario, current clinical stents are either DES or bio-resorbable stents which, in either case, are not conductive and hence would not present any offset or conductive issues for measurements. Since the strut thickness is relatively small, the sizing measurements is essentially that of lumen area or MSA. Furthermore, although the measurements were made on a conductance catheter, the methodology presented is completely translatable to a sizing guidewire as shown in peripheral arteries of animals and patients (Svendsen et al., 2014b; Nair et al., 2018). For example, our peripheral guidewire can size the Supera (Abbott) or the Viabahn (Gore) as neither of these devices is electrically conductive.

### Limitations of Study

As with any technology, the conductance method has limitations. First, the electrical conductivity measurements of lumen size cannot confirm stent apposition (i.e., this method is non-tomographic and hence does not allow visualization of relation between struts and vessel wall at the current state). A sizing post-dilation method using the same electrical platform technology can be used to address this issue for coronary or peripheral applications (Svendsen et al., 2014a, 2015). Second, the saline injections (although routine in the clinic) do add steps to the procedure. An injection-less method is possible with the current technology where feasibility has been recently demonstrated (Dabiri and Kassab, 2018). Finally, the comparison of accuracy is made in comparison with IVUS. Ultimately, a clinical outcome study may be necessary to establish the clinical utility of this technology similar to IVUS studies (Zhang et al., 2018).

## Summary

We validated a conductance device that allows accurate sizing of the stent vessel. Undoubtedly, a workhorse guidewire that allows reliable and accurate assessment of coronary and peripheral stent area may provide a powerful treatment tool for the interventionalist. This may improve clinical outcomes by ensuring the desired MSA without over-distension which should lead to better clinical outcome.

## Author Contributions

The author confirms being the sole contributor of this work and has approved it for publication.

## Conflict of Interest Statement

GK is founder of 3DT Holdings.
